# Role of Fenugreek, Cinnamon, *Curcuma longa*, Berberine and *Momordica charantia* in Type 2 Diabetes Mellitus Treatment: A Review

**DOI:** 10.3390/ph16040515

**Published:** 2023-03-30

**Authors:** Marisol Cortez-Navarrete, Karina G. Pérez-Rubio, Miriam de J. Escobedo-Gutiérrez

**Affiliations:** Institute of Experimental and Clinical Therapeutics, Department of Physiology, Health Science University Center, University of Guadalajara, Sierra Mojada 950, Col. Independencia, Guadalajara 44340, Jalisco, Mexico; karina.prubio@academicos.udg.mx (K.G.P.-R.); miriam.escobedo3107@alumnos.udg.mx (M.d.J.E.-G.)

**Keywords:** berberine, cinnamon, *Curcuma longa*, fenugreek, medicinal plant, *Momordica charantia*, type 2 diabetes mellitus

## Abstract

Type 2 diabetes mellitus (T2DM) is a complex disease that has become a major global health concern. Given the efficacy of antidiabetic drugs, pharmacological therapy is considered the first-line treatment of T2DM; however, due to their potential side effects and high costs, new and cost-effective treatments with minimal side effects are needed. Medicinal plants have been used for centuries as part of traditional medicine to treat T2DM. Among these, fenugreek, cinnamon, *Curcuma longa*, berberine, and *Momordica charantia* have demonstrated different degrees of hypoglycemic activity in clinical studies and animal models. Therefore, the aim of this review is to synthesize the mechanisms of action of five medicinal plants, as well as the experimental and clinical evidence of their hypoglycemic activity from the published literature.

## 1. Introduction

Diabetes mellitus is the collective term for heterogeneous metabolic disorders characterized by chronic hyperglycemia [1]. Type 2 diabetes mellitus (T2DM) is the most common form of the disease, which is characterized by relative insulin secretion deficiency and insulin resistance in target organs [2]. Moreover, T2DM is a complex and chronic condition that requires continuous medical care focused on multifactorial risk-reduction strategies [3]. Currently, there are many drugs available for the treatment of T2DM; however, they are associated with a high incidence of side effects including hypoglycemia, diarrhea, nausea, vomiting, abdominal pain, and weight fluctuation, among others. In addition to the unwanted side effects of synthetic drugs, their high costs, unaffordability, lack of equitable distribution, and low compliance emphasize the need for alternative therapies [4,5,6]. Recently, many medicinal plants have demonstrated potential for the treatment of T2DM because of their high content of bioactive compounds such as phenolics, glycosides, alkaloids, steroidal saponins, terpenoids, flavonoids, and carotenoids, which may possess antidiabetic activities [7].

A significant number of medicinal plants have demonstrated different degrees of hypoglycemic activity in clinical studies and animal models [8,9,10,11,12,13,14,15,16,17,18,19,20,21,22,23,24,25,26,27,28,29,30,31,32]. Therefore, the aim of this review is to synthesize the experimental and clinical evidence, including the mechanisms of action of five medicinal plants with hypoglycemic effects: fenugreek, cinnamon, *Curcuma longa*, berberine, and *Momordica charantia*.

## 2. Medicinal Plants for the Treatment of Type 2 Diabetes Mellitus

### 2.1. Fenugreek

Fenugreek (*Trigonella foenum-graecum*) is an annually grown herb that belongs to the family Fabaceae. It is cultivated in India, northern Africa, South Asia, Europe, Australia, and Argentina [33]. Fenugreek seeds are known for their medicinal properties and are used as a spice worldwide, whereas leaves are used in culinary preparations in several countries [34]. Fenugreek seeds are rich in soluble dietary fiber and protein and contain various types of alkaloids, flavonoids, and saponins. The most studied bioactive compounds are diosgenin, 4-hydroxyisoleucine, and trigonelline. Fenugreek also contains ascorbic acid, retinol, β-carotene, thiamine, riboflavin, and folic acid [33,35].

**Figure 1:**
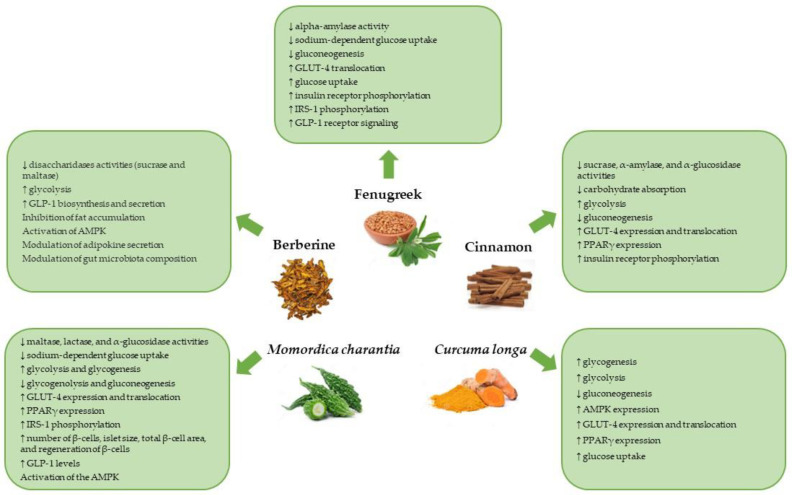
Mechanisms of action behind the hypoglycemic effects of fenugreek, cinnamon, *Curcuma longa*, berberine, and *Momordica charantia*. GLUT-4: glucose transporter 4, IRS-1: insulin receptor substrate 1, GLP-1: glucagon-like peptide-1, PPARγ: peroxisome proliferator-activated receptor gamma, AMPK: adenosine monophosphate-activated protein kinase. ↑: increase, ↓: decrease.

Fenugreek is a commonly used herb for the management of T2DM. The antidiabetic effect of fenugreek is associated with different mechanisms of action (Figure 1). In an in vitro study, Fenugreek seed extract inhibited alpha-amylase activity in a dose-dependent manner. Suppression of starch digestion and absorption induced by fenugreek seed extract was confirmed in normal rats in the same study [36]. The hypoglycemic effect of fenugreek is also associated with the inhibition of glucose uptake. Fenugreek seed extract inhibited intestinal sodium-dependent glucose uptake in vitro in rabbit intestinal brush border membrane vesicles [37].

A secondary mechanism by which fenugreek may regulate plasma glucose levels is by delaying gastric emptying and slowing down the rate of post-prandial glucose absorption due to its high soluble fiber content [8,38]. It has been shown that fenugreek can also regulate the activity of key regulatory gluconeogenic enzymes. In streptozotocin (STZ)-induced diabetic rats, administration of Fenugreek seed extract decreased liver glucose-6-phosphatase activity, thus suppressing hepatic gluconeogenesis [36]. Treatment of alloxan-induced diabetic rats with fenugreek whole seed powder also reduced high renal and hepatic glucose-6-phosphatase and fructose-1,6-bisphosphatase values to normal levels [39].

Scientific evidence suggests insulinotropic activity by 4-hydroxyisoleucine, a free amino acid present in fenugreek seeds. Results from in vitro experiments showed that 4-hydroxyisoleucine increased insulin secretion through a direct effect on both isolated rat and human islets of Langerhans [40]. A previous experiment in rats also reported that 4-hydroxyisoleucine stimulated insulin secretion [41].

Regulation of other pancreatic hormones is also a possible mechanism of the antidiabetic effects of fenugreek. The administration of fenugreek seed extract to alloxan-induced diabetic dogs reduced glucagon and somatostatin levels [42].

Furthermore, fenugreek may play a role in regulating glucagon-like peptide-1 (GLP-1) activity. It has been shown that an active compound (N55), isolated from fenugreek seeds, binds to GLP-1 and enhances the potency of GLP-1 in stimulating GLP-1 receptor signaling [43].

Activation of an insulin signaling pathway in adipocytes and liver cells has also been reported. Fenugreek seed extract stimulated tyrosine phosphorylation of the insulin receptor, insulin receptor substrate 1 (IRS-1), and the p85 subunit of phosphatidylinositol 3-kinase (PI3K) in both 3T3-L1 adipocytes and human hepatoma cells. Fenugreek seed extract also stimulated glucose uptake in CHO-HIRc-myc-GLUT4eGFP cells. This effect was shown to be mediated by the translocation of glucose transporter 4 (GLUT-4) from the intracellular space to the plasma membrane [44]. Moreover, fenugreek can improve insulin sensitivity in skeletal muscle cells and adipocytes. 4-hydroxyisoleucine promoted glucose uptake in L6-GLUT4myc myotubes by enhancing translocation of GLUT-4 to the cell surface in a PI3K/protein kinase B-dependent pathway [45]. Treatment of L6 myotubes with 4-hydroxyisoleucine restored insulin sensitivity by regulating the function of IRS-1 [46]. Similar findings were observed in 3T3-L1 adipocytes, where 4-hydroxyisoleucine restored insulin sensitivity and attenuated tumor necrosis factor-alpha (TNF-alpha) expression in response to palmitate-induced insulin resistance [47].

Several animal experimental models have also demonstrated the hypoglycemic effect of fenugreek. Administration of a soluble dietary fiber fraction of Fenugreek to STZ-induced type 2 diabetic rats (0.5 g/kg body weight) suppressed the elevation of blood glucose after oral sucrose ingestion, improving glucose tolerance. Longer-term administration of fenugreek for 28 days also reduced fasting serum glucose and increased liver glycogen content [8].

In STZ-induced diabetic rats, fenugreek administration (0.44, 0.87, and 1.74 g/kg/day) for 6 weeks significantly decreased fasting blood glucose and glycated hemoglobin levels compared to untreated diabetic rats [9]. STZ-induced diabetic rats treated with fenugreek seed extract (50, 100, and 200 mg/kg) for 6 weeks significantly reduced blood glucose levels in all extract-treated groups compared to the diabetic group [48]. Oral treatment with 4-hydroxyisoleucine (100 mg/kg) to STZ-induced diabetic rats lowered blood glucose levels and improved blood glucose impairment as evaluated by an oral glucose tolerance test (OGTT). In addition, treatment with 4-hydroxyisoleucine upregulated insulin expression in pancreatic tissue [49]. Another study in STZ-induced diabetic rats reported an increase in serum insulin levels and a significant decrease in serum glucose levels after oral administration of fenugreek extract (0.1, 0.25, and 0.5 g/kg body weight) for 14 days [50].

A large number of clinical studies have evaluated the antidiabetic effect of fenugreek in T2DM patients (Table 1). Rafraf et al. conducted a randomized, triple-blind, placebo-controlled clinical trial in 88 T2DM patients. Subjects were randomly allocated to receive powdered whole Fenugreek seeds (10 g/day) or a placebo for 8 weeks. Fasting plasma glucose (FPG) levels (*p* = 0.007), glycated hemoglobin A1c (A1C) levels (*p* = 0.0001), and the homeostasis model assessment of insulin resistance (HOMA-IR) (*p* = 0.004) significantly decreased in the fenugreek group compared to the placebo group [10]. Verma et al. carried out a randomized, double-blind, placebo-controlled clinical trial in 154 patients with T2DM. Subjects were randomly assigned to fenugreek seed extract (1 g/day) or a placebo for 90 days in addition to standard antidiabetic therapy (metformin). A patented fenugreek seed extract (FenfuroTM), enriched in approximately 40% furostanolic saponins, was used in the study. FPG levels (151.31 ± 24.42 mg/dL vs. 118.05 ± 25.33 mg/dL, *p* = 0.000) and postprandial plasma glucose (PPG) levels (251.01 ± 68.88 mg/dL vs. 174.78 ± 54.90 mg/dL, *p* = 0.000) significantly decreased after fenugreek administration. However, the reduction in A1C was not statistically significant [11].

Ranade et al. evaluated the effect of fenugreek in a randomized, single-blind, parallel-group clinical study. Sixty patients with T2DM who were treated with any antidiabetic medication (oral hypoglycemic agent, insulin) were included in the study. Patients were randomized to receive fenugreek seeds soaked in hot water (10 g/day) for 6 months, while patients in the other group only received antidiabetic medication. Significant changes in FPG levels (fenugreek group: 122.21 ± 23.25 mg/dL; control group: 153.59 ± 23.37 mg/dL; *p* = 0.0351) and A1C levels (fenugreek group: 5.78 ± 1.56%; control group: 6.96 ± 2.02%; *p* = 0.0201) were observed between the fenugreek group and the control group [12].

Gupta et al. performed a double-blind, placebo-controlled clinical trial to study the effect of fenugreek administration in 25 newly diagnosed T2DM patients. Patients were assigned to receive a hydroalcoholic extract of fenugreek seeds (1 g/day) or a placebo for 2 months. Oral hypoglycemic agents were permitted if blood glucose control was considered inadequate (a combination of sulfonylurea and biguanides). FPG levels (148.3 ± 44.1 mg/dL vs. 119.9 ± 25 mg/dL, *p* < 0.05) significantly decreased compared to baseline values in the fenugreek group. A significant increase in percent insulin sensitivity determined by the HOMA model (57.14 ± 41.15% vs. 112.9 ± 67%, *p* < 0.05) was also reported among patients who received fenugreek [51].

Najdi et al. conducted a randomized, open-label clinical study in T2DM patients to evaluate the hypoglycemic effect of fenugreek compared to glibenclamide. The study included 12 patients treated with conventional therapy (metformin) who received fenugreek seed (2 g/day, GNC company) or glibenclamide for 12 weeks. A significant increase in fasting insulin levels (6.98 ± 1.78 μU/mL vs. 9.62 ± 3.16 μU/mL, *p* = 0.04) was observed in the fenugreek group. There were no significant changes in FPG, A1C, and HOMA-IR after the administration of fenugreek [52].

Lu et al., assessed the effect of fenugreek administration in combination with sulfonylureas in 69 T2DM patients. In this randomized, double-blind, placebo-controlled clinical study, patients received either fenugreek saponin extract (0.35 g/pill; each gram of powder equals to 16 g of crude drug, 6 capsules each time and thrice a day) or a placebo for 12 weeks. Significant decreases from baseline to endpoint were reported in FPG levels (8.63 ± 1.71 mmol/L vs. 6.79 ± 1.40 mmol/L, *p* < 0.05), PPG levels (13.34 ± 3.99 mmol/L vs. 9.46 ± 2.17 mmol/L, *p* < 0.01), and A1C (8.02% ± 1.67% vs. 6.56% ± 0.95%, *p* < 0.05) among patients who received fenugreek. When compared to the placebo group, FPG levels, PPG levels, and A1C also reduced significantly in the fenugreek group (*p* < 0.05, *p* < 0.01, and *p* < 0.05, respectively) [53].

Sharma et al., conducted a randomized, crossover-design clinical study in 15 T2DM subjects treated with antidiabetic drugs (glibenclamide, glipizide, or metformin). Patients were randomized to receive fenugreek seed powder (100 g/day) incorporated into chapati (unleavened bread) for 10 days, while the rest received the same in the second period. FPG levels (179.00 ± 24.00 mg/dL vs.137.00 ± 20.20 mg/dL, *p* < 0.05) and PPG levels (402.00 ± 43.00 mg/dL vs. 292.00 ± 33.70 mg/dL, *p* < 0.01) significantly decreased compared to baseline values in the fenugreek group. The effect of the same intervention for 20 days was assessed in a subset of the study. Significant reductions in FPG levels (157.00 ± 22.20 mg/dL vs.116.00 ± 17.10 mg/dL) and PPG levels (*p* < 0.05) were also reported after the intervention with fenugreek [54].

The effect of fenugreek administration in prediabetes was reported by Gaddam et al. A randomized, single-blind, controlled, parallel-group clinical study was carried out to evaluate if the long-term intake of fenugreek can prevent the outcome of T2DM in patients with prediabetes. Patients were divided into two groups: one group received fenugreek seed powder (10 g/day) for 3 years, and the other group served as the control. The study reported a significant reduction in FPG levels (103.7 ± 9.5 mg/dL vs. 99.7 ± 11.4 mg/dL, *p* < 0.05) and PPG levels (142.9 ± 26.6 mg/dL vs. 129.0 ± 29.6 mg/dL, *p* < 0.01) in the fenugreek group; whereas serum insulin levels (10.2 ± 4.5 mU/L vs. 12.0 ± 5.6 mU/L, *p* < 0.01) significantly increased after fenugreek administration. Supplementation with fenugreek was associated with a lower progression to diabetes as the conversion rate from impaired fasting glucose and impaired glucose tolerance (IGT) at the end of a 3-year period significantly decreased in the fenugreek group when compared to controls [55].

**Table 1:** Fenugreek: clinical evidence in type 2 diabetes mellitus.

Medicinal Plant	Sex/Age (Range or Mean)	T2DM Inclusion Criteria	Blinding/ Randomization	Intervention	Results	Reference
Fenugreek (powdered whole seeds)	Women and men 40.535 years	Diagnostic criteria not reported	Triple-blind/randomized (method not reported)	10 g/day for 8 weeks	↓ FPG ↓ A1C ↓ HOMA-IR	[10]
Fenugreek (seed extract)	Women and men 25–60 years	FPG ≤ 180 mg/dL A1C > 7.5%	Double-blind/randomized (computer-generated randomization code)	1 g/day for 90 days	↓ FPG ↓ PPG ↔ A1C	[11]
Fenugreek (seeds soaked in hot water)	Women and men >18 years	American Diabetes Association: FPG ≥ 126 mg/dL A1C ≥ 6.5% 2-h PG during OGTT ≥ 200 mg/dL	Single-blind/randomized (Stat Trek’s Random Number Generator)	10 g/day for 6 months	↓ FPG ↓ A1C	[12]
Fenugreek (hydroalcoholic seed extract)	Women and men 49.16 ± 6.57 years	FPG < 200 mg/dL	Double-blind/randomization not reported	1 g/day for 2 months	↓ FPG ↑ insulin sensitivity	[51]
Fenugreek (seed)	Women and men >18 years	FPG ≥ 140 mg/dL A1C ≥ 7%	Open-label/randomized (computer-generated randomization list)	2 g/day for 12 weeks	↑ fasting insulin ↔ FPG ↔ A1C ↔ HOMA-IR	[52]
Fenugreek (saponin extract)	Women and men 25–65 years	American Diabetes Association: FPG > 7 mmol/L and <13 mmol/L	Double-blind/randomized (random number table in the ratio of 2:1)	0.35 g/pill; each gram of powder equals to 16 g of crude drug, 6 capsules each time and thrice a day for 12 weeks	↓ FPG ↓ PPG ↓ A1C	[53]
Fenugreek (seed powder)	Women and men 32–60 years	World Health Organization criteria	Blinding not reported/randomized (method not reported)	100 g/day for 10 or 20 days	↓ FPG ↓ PPG	[54]
Fenugreek (seed powder)	Women and men 30–70 years	FPG ≥ 100 mg/dL and ≤125 mg/dL 2-h PG during OGTT ≥ 140 mg/dL and ≤199 mg/dL	Single-blind/randomized (computer-generated random numbers)	10 g/day for 3 years (prediabetic patients)	↓ FPG ↓ PPG ↑ fasting insulin	[55]

T2DM: type 2 diabetes mellitus; FPG: fasting plasma glucose; A1C: glycated hemoglobin A1c; HOMA-IR: homeostasis model assessment of insulin resistance; PPG: postprandial plasma glucose; PG: plasma glucose; OGTT: oral glucose tolerance test. Parameter changes: ↓: significant decrease; ↑: significant increase; ↔: unchanged.

### 2.2. Cinnamon

The genus *Cinnamomum* comprises about 300 species, of which Ceylon Cinnamon (*C. zeylanicum*) and Chinese Cinnamon (*C. cassia*) are the most widely studied for their beneficial health effects on health [56]. *C. zeylanicum*, comes from the tree of the Lauraceous family and is native to Sri Lanka, India, Madagascar, and South America. The spice has a fragrant aroma and a sweet flavor and is made up of the inner dried bark of the tree [57,58]. *C. cassia*, obtained from the cassia tree, is cultivated in Asia, especially in southern China and northern Vietnam [59]. It is also used as a spice and is also made up of the inner tree bark and has a strong spicy-sweet flavor [59,60].

Many bioactive compounds have been found in cinnamon, such as polyphenolic compounds [61]. The main active components of *C. zeylanicum* are cinnamaldehyde, cinnamyl acetate, β-caryophyllene, α-terpineol, eugenol, and proanthocyanidin cinnammtannin B1 [57,61,62]. The main active compounds of C. cassia are cinnamaldehyde, cinnamic acid, cinnamyl alcohol, coumarin lignans, and phenylpropanoids [60].

Scientific evidence indicates that cinnamon administration has therapeutic effects, such as antidiabetic and insulin-sensitizing activity through several mechanisms of action (Figure 1). It has been reported that in an in vitro model *C. zeylanicum* bark extract can inhibit intestinal sucrase, pancreatic α-amylase, and α-glucosidase activities, and thus reduce carbohydrate digestion and absorption [63,64,65]. Inhibition of pancreatic α-amylase activity was also observed in diabetic rats treated with *C. zeylanicum* [58,66]. Cinnamaldehyde (from *C. zeylanicum*) reduced the activity of phosphoenolpyruvate carboxykinase (PEPCK) and normalized PEPCK messenger RNA (mRNA) levels in the liver and kidney of diabetic rats. This enzyme is key in the gluconeogenic pathway [13]. Cinnamaldehyde also increased the activity of the glycolytic enzyme pyruvate kinase in the liver tissue of diabetic rats [13]. It should be noted that the increased activity of glucose-6-phosphatase and fructose-1,6-bisphosphatase is associated with a gluconeogenic state. In fructose-fed-rats, the administration of *C. zeylanicum* bark extract reduced the activity of these enzymes [67]. In 3T3-L1 adipocytes, proanthocyanidin cinnammtannin B1 (from *C. zeylanicum* bark extract) activated the phosphorylation of insulin receptors through the activation of the PI3K cascade [62].

It has also been reported that *C. zeylanicum* can stimulate GLUT-4 production and translocation to the cell membrane in skeletal muscle and brown adipose tissue of diabetic rats [68]. A similar effect was observed with cinnamaldehyde (from *C. zeylanicum*) in adipocyte cell lines and diabetic rats [13]. In addition, the administration of cinnamaldehyde (from *C. zeylanicum*) to diabetic rats significantly increased glycogen in liver and muscle tissue [13,69]. Significantly increased mRNA expression levels of peroxisome proliferator-activated receptor gamma (PPARγ) on adipose tissue were found in diabetic mice treated with *C. cassia* bark extract, thereby improving insulin sensitivity [70].

Numerous experiments in animal models have also evaluated the hypoglycemic potential of cinnamon and its bioactive compounds. The administration of cinnamaldehyde (from *C. zeylanicum*) in STZ-induced diabetic rats (20 mg/kg) for 60 days significantly reduced FPG and A1C levels compared to the untreated diabetic control group; insulin levels also significantly increased compared to the untreated diabetic control group and the cinnamaldehyde intervention group [13]. Similar results were observed after the administration of *C. zeylanicum* (group I: 0.5 g and group II: 1 g) in STZ-induced diabetic rats. A significant decrease in FPG levels and a significant increase in insulin levels were observed in both cinnamon groups compared to the diabetic group [14].

STZ-induced diabetic rats treated with water extract of *C. zeylanicum* sticks (3 mg/kg, 30 mg/kg, and 100 mg/kg) for 22 days significantly reduced FPG levels in rats with 30 mg/kg and 100 mg/kg cinnamon treatment compared to the diabetic group [68]. Treatment with *C. cassia* extract powder (200 mg/kg) for 12 weeks in C57BL/Ks db/db mice significantly decreased FPG and PPG levels and significantly increased insulin levels compared to the diabetic control group [70]. Another study in STZ-induced diabetic rats reported a significant reduction in FPG levels and a significant increase in insulin levels after the administration of ethanolic bark extract of *C. cassia* (500 mg/kg) for 28 days compared to the diabetic group [71].

Several clinical trials have evaluated the antidiabetic effects of cinnamon in T2DM patients (Table 2). A study conducted by Khan et al. was the first that reported a favorable effect of *C. cassia* on FPG levels in a randomized, placebo-controlled clinical trial of 60 T2DM patients with sulfonylurea drugs as base treatment. Patients were randomly assigned to three single oral doses of *C. cassia* (1, 3, or 6 g/day) or a placebo for 40 days. After cinnamon intake, all three study groups significantly decreased FPG levels (group 1 g/day: 11.6 ± 1.7 mmol/L vs. 8.7 ± 1.6 mmol/L, *p* < 0.05; group 3 g/day: 11.4 ± 1.2 mmol/L vs. 9.4 ± 1.1 mmol/L, *p* < 0.05; group 6 g/day: 13.0 ± 1.4 mmol/L vs. 9.2 ± 1.5 mmol/L, *p* < 0.05) [15]. Similar results were observed by Radhia et al., in a randomized, placebo-controlled study after the administration of *C. cassia* (1.5 g/day) or a placebo for 30 days to 14 T2DM patients. At the end of the intervention, a significant decrease was observed in FPG levels (216.3 ± 52.7 mg/dL vs. 163.3 ± 44.9 mg/dL, *p* < 0.05) in the cinnamon group [16].

Akilen et al. carried out a randomized, double-blind, placebo-controlled clinical trial on 58 T2DM patients treated with oral hypoglycemic agents. Subjects were randomly allocated to receive *C. cassia* dried bark powder (2 g/day) or a placebo for 12 weeks. A significant reduction was observed in A1C levels (cinnamon group: 7.86 ± 1.42%; control group: 8.68 ± 1.83%, *p* = 0.029) in the cinnamon group compared to the placebo group. Significant decreases were also observed in the cinnamon group in A1C levels (8.22 ± 1.16% vs. 7.86 ± 1.42%, *p* < 0.05) and FPG levels (8.82 ± 3.45 mmol/L vs. 8.04 ± 3.10 mmol/L, *p* < 0.05) [17].

Vafa et al., performed a randomized, double-blind, placebo-controlled study on 44 T2DM patients. The participants were randomly allocated to receive *C. zeylanicum* (3 g daily) or a placebo for 8 weeks. All subjects had a stable medication regimen over the last month (metformin or gliclazide). Significant reductions compared to baseline values were observed in FPG levels (139.28 ± 9.11 mg/dL vs. 126.47 ± 17.73 mg/dL, *p* = 0.005) and A1C levels (7.35 ± 0.51% vs. 6.9 ± 0.77%, *p* = 0.008) in the cinnamon group. However, insulin levels were not significantly modified after the administration of cinnamon [72]. Lu et al. evaluated the effect of two different doses of *C. cassia* extract in a randomized, double-blind, placebo-controlled clinical trial on 69 T2DM patients with gliclazide as a stable treatment. Patients were randomly allocated to receive *C. cassia* extract (120 mg/day or 360 mg/day) or a placebo for 3 months. At the end of the intervention period, significant decreases were observed in both cinnamon groups in their FPG levels (120 mg/day group: 9.00 ± 1.23 mmol/L vs. 7.99 ± 1.05 mmol/L, *p* = 0.002 and 360 mg/day group: 11.21 ± 2.21 mmol/L vs. 9.59 ± 1.66 mmol/L, *p* = 0.00008) and A1C levels (120 mg/day group: 8.90 ± 1.24% vs. 8.23 ± 0.99%, *p* = 0.003 and 360 mg/day group: 8.92 ± 1.35% vs. 8.00 ± 1.00%, *p* = 0.0004) [73].

Anderson et al. conducted a randomized, double-blind, placebo-controlled study on 173 patients with elevated serum glucose (FPG > 6.1 mmol/L and <20 mmol/L or 2-h glucose > 7.8 mmol/L and <25 mmol/L) and without insulin therapy. Subjects were randomly assigned to receive a water extract of *C. cassia* (500 mg/day, CinSulin^®^) or a placebo for 2 months. Significant decreases from baseline to endpoint were reported in FPG levels (8.85 ± 0.36 mmol/L vs. 8.19 ± 0.29 mmol/L, *p* < 0.005), PPG levels (15.09 ± 0.57 mmol/L vs. 13.30 ± 0.55 mmol/L, *p* < 0.0001), and HOMA-IR (9.67 ± 0.90 vs. 8.32 ± 0.84, *p* < 0.005) among patients who received *C. cassia* [74].

Zare et al. assessed the effect of *C. zeylanicum* administration on 140 T2DM patients with only hypoglycemic agents as base treatment. In this randomized, triple-blind, placebo-controlled clinical trial, patients were randomly allocated to receive *C. zeylanicum* (1000 mg/day) or a placebo for 3 months based on their body mass index (BMI) (two groups with cinnamon treatment, group I: BMI ≥ 27 and group II: BMI < 27, and two groups with a placebo, group III: BMI ≥ 27 and group IV: BMI < 27). A significant change was observed from baseline to endpoint between the cinnamon and placebo groups in patients with BMI ≥ 27 in FPG levels (cinnamon group: −19.37 ± 2.3 mg/dL; placebo group: −0.22 ± 1.53 mg/dL, *p* < 0.001), PPG levels (cinnamon group: −21.35 ± 3.8 mg/dL; placebo group: 1.97 ± 6.5 mg/dL, *p* = 0.003), A1C levels (cinnamon group: −0.42 ± 0.06%; placebo group: 0.044 ± 0.01%, *p* < 0.001), and HOMA-IR (cinnamon group: −1.41 ± 0.16; placebo group: −0.004 ± 0.05, *p* < 0.001). In addition, a significant change was observed in patients with BMI < 27 in FPG levels (cinnamon group: −11.9 ± 2.7 mg/dL; placebo group: −9.5 ± 5.4 mg/dL, *p* = 0.03) and HOMA-IR (cinnamon group: −0.55 ± 0.10; placebo group: −0.06 ± 0.03, *p* < 0.001) [75].

Talaei et al. evaluated the effect of *C. zeylanicum* (3 g/day) on the glycemic control parameters of 44 T2DM patients with metformin therapy in a randomized, double-blind, placebo-controlled study. The duration of the intervention was 8 weeks. The study reported no significant changes in the cinnamon group and at the end of intervention in FPG levels (183.85 ± 36.16 mg/dL vs. 172.20 ± 44.86 mg/dL, *p* = 0.09), A1C levels (10.04 ± 1.30% vs. 10.11 ± 1.49%, *p* = 0.83), fasting insulin levels (9.85 (7.92–19.22) mU/L vs. 12.10 (10.65–18.45) mU/L, *p* = 0.24), and HOMA-IR (5.35 (2.97–9.22) vs. 6.00 (3.34–9.00), *p* = 1.00). In addition, no significant differences were identified compared to the placebo group in FPG, A1C, fasting insulin levels, and HOMA-IR [76].

**Table 2:** Cinnamon: clinical evidence in type 2 diabetes mellitus.

Medicinal Plant	Sex/Age (Range or Mean)	T2DM Inclusion Criteria	Blinding/ Randomization	Intervention	Results	Reference
Cinnamon from *C. cassia*	Women and men >40 years	FPG ≥ 7.8 mmol/L and ≤22.2 mmol/L	Blinding not reported/randomized (block randomization)	1, 3 or 6 g/day for 40 days	↓ FPG	[15]
Cinnamon from *C. cassia*	Women and men ≥40 years	FPG > 125 mg/dL	Blinding not reported/randomized (method not reported)	1.5 g/day for 30 days	↓ FPG	[16]
Cinnamon from *C. cassia* (dried bark powder)	Women and men ≥18 years	FPG > 7 mmol ⁄ L A1C > 7%	Double-blind/randomized (computer generated randomized list)	2 g/day for 12 weeks	↓ FPG ↓ A1C	[17]
Cinnamon from *C. zeylanicum*	Women and men 30–65 years	World Health Organization: FPG ≥ 126 mg/dL and ≤160 mg/dL A1C ≥ 6% and ≤8%	Double-blind/randomized (method not reported)	3 g/day for 8 weeks	↓ FPG ↓ A1C ↔ fasting insulin	[72]
Cinnamon from *C. cassia* (extract)	Women and men >48 years	FPG > 8.0 mmol/L A1C > 7.0%	Double-blind/randomized (method not reported)	120 mg/day or 360 for 3 months	↓ FPG ↓ A1C	[73]
Cinnamon from *C. cassia* (water extract)	Women and men 61.3 ± 0.8 years	FPG > 6.1 mmol/L 2-h PG during OGTT > 7.8 mmol/L	Double-blind/randomized (random number table)	500 mg/day for 2 months (elevated serum glucose patients)	↓ FPG ↓ PPG ↓ HOMA-IR	[74]
Cinnamon from *C. zeylanicum*	Women and men 30–80 years	American Diabetes Association: FPG ≥ 126 mg/dL and ≤250 mg/dL	Triple-blind/randomized (random number table)	1000 mg/day for 3 months	↓ FPG ↓ PPG ↓ A1C ↓ HOMA-IR	[75]
Cinnamon from *C. zeylanicum*	Women and men 25–70 years	FPG < 180 mg/dL 2-h PG during OGTT < 250 mg/dL	Double-blind/randomized (method not reported)	3 g/day for 8 weeks	↔ FPG ↔ A1C ↔ fasting insulin ↔ HOMA-IR	[76]

T2DM: type 2 diabetes mellitus; FPG: fasting plasma glucose; A1C: glycated hemoglobin A1c; PPG: postprandial plasma glucosa; HOMA-IR: homeostasis model assessment of insulin resistance; PG: plasma glucose; OGTT: oral glucose tolerance test. Parameter changes: ↓: significant decrease; ↑: significant increase; ↔: unchanged.

### 2.3. Curcuma longa

*Curcuma longa*, also known as turmeric, is a tuberous herbaceous perennial plant that belongs to the ginger family, which is characterized by orange tuberous rhizomes. It grows in tropical climates and is found in southern and southwest Asia. This plant is used in the cuisines of Iran, Malesia, India, China, Polynesia, and Thailand to add flavor and color to different meals. *Curcuma longa* is widely used in Ayurveda and traditional Chinese medicine [77].

*Curcuma longa* contains carbohydrates, protein, fat, moisture, and minerals [77]. Many bioactive compounds known as curcuminoids have been found in *Curcuma longa* rhizomes. The most important curcuminoids are curcumin, demethoxycurcumin, and bisdemethoxycurcumin [78].

The most active curcuminoid, curcumin, exerts antidiabetic effects through different mechanisms of action described in vitro and in animal model studies (Figure 1).

Curcumin administration increased adenosine monophosphate-activated protein kinase (AMPK) expression in the liver of db/db mice, which inhibits hepatic glucose production [79]. In db/db mice, treatment with curcumin increased glycogen storage in the liver and the activity of hepatic glucokinase, a glycolytic enzyme, while it inhibited glucose-6-phosphatase and PEPCK activities, which are both gluconeogenic enzymes [19].

Curcumin treatment in a high-fat diet (HFD) plus STZ-induced diabetic rats and insulin-resistant L6 myotubes enhanced glycogen synthesis by decreasing the phosphorylation of glycogen synthase [80]. In the same study, treatment with curcumin inhibited the expression of pyruvate dehydrogenase kinase 4 (PDK4) in both diabetic rats and L6 myotubes. PDK4 phosphorylates and deactivates pyruvate dehydrogenase, which is an important enzymatic complex involved in glucose oxidation [80]. An increase in GLUT-4 expression and translocation to cell membranes were also observed in insulin-resistant L6 myotubes treated with curcumin [80].

In addition, curcumin enhanced hepatic peroxisome proliferator-activated receptor gamma PPARγ in the liver of db/db mice, which is linked to glucose homeostasis and insulin sensitivity [79,81]. In another study, curcumin activated M-1 cholinergic receptors and increased glucose uptake through the phospholipase C/PI3K pathway in the skeletal muscle of Wistar rats [82].

Numerous experimental studies in animal models have demonstrated the hypoglycemic effect of curcumin. After the administration of curcumin dissolved in olive oil (100 mg/kg) to STZ-induced diabetic rats for 7 weeks, significant reductions in FPG and A1C levels were observed compared to diabetic rats treated only with olive oil [18]. In C57BL/KsJ db/db mice, curcumin administration (0.2 g/kg) for 6 weeks significantly reduced the FPG levels, A1C levels, and HOMA-IR compared to untreated diabetic mice. Furthermore, treatment with curcumin significantly increased insulin levels compared to the control group [19]. STZ-induced diabetic rats treated with curcumin (50, 150, or 250 mg/kg) for 7 weeks significantly decreased FPG levels with 150 and 250 mg/kg of curcumin treatment and PPG levels in all curcumin-treated groups compared to the diabetic group [80].

Treatment with curcumin (80 mg/kg/day) to HFD-induced T2DM rats for 75 days, significantly lowered FPG levels, OGTT levels, and HOMA-IR compared to the untreated diabetic group [83]. However, no significant changes were observed in blood glucose levels in diabetic db/db mice after administration of a diet supplemented with curcumin (0.75%) for 8 weeks compared to the control group [79].

Several clinical trials have evaluated the antidiabetic effect of *Curcuma longa* and its curcuminoids in T2DM (Table 3). Na et al., conducted a randomized, double-blind, placebo-controlled study. One hundred nine overweight/obese T2DM patients were randomly assigned to a combination of curcuminoids (300 mg/day: curcumin, demethoxycurcumin, and bisdemethoxycurcumin) or a placebo for 3 months in addition to standard antidiabetic therapy (hypoglycemic agents, insulin or both). FPG levels (7.28 ± 1.77 mmo/L vs. 8.17 ± 2.06 mmol/L, *p* < 0.01), A1C levels (7.02 ± 2.04% vs. 7.99 ± 2.86%, *p* = 0.031), and HOMA-IR (4.14 ± 1.81 vs. 5.49 ± 2.15, *p* < 0.01) significantly decreased in the curcuminoid group compared to the placebo group [20].

Rahimi et al. carried out a randomized, double-blind, placebo-controlled clinical trial on 70 T2DM patients. Patients were assigned to curcumin in nano-micelles (80 mg/day) or a placebo for 3 months. A registered curcumin product (SinaCurcumin^®^) was used in this clinical trial. At the endpoint, significant reductions in FPG levels (135.5 ± 51.33 mg/dL vs. 120.29 ± 38.01 mg/dL, *p* = 0.049) and A1C levels (7.59 ± 1.74% vs. 7.31 ± 1.54%, *p* < 0.001) were observed in the curcumin group. The study also reported a significant decrease in FPG levels (curcumin group: 120.29 ± 38.01 mg/dL; placebo group: 176.0 ± 61.56 mg/dL, *p* = 0.004) and A1C levels (curcumin group: 7.31 ± 1.54%; placebo group: 9.00 ± 2.33%, *p* = 0.02) at the end of the study in the curcumin group compared to the placebo group [21].

Asadi et al. evaluated the effect of nano-curcumin in a randomized, double-blind, placebo-controlled study. In this study, 80 non-insulin dependent T2DM patients were randomly allocated to nano-curcumin (80 mg/day) or a placebo for 8 weeks. Nano-curcumin from Exir Nano Sina Company was used in the study. Significant changes in FPG levels (nano-curcumin group: 150.9 ± 58.1 mg/dL; placebo group:189.7 ± 62.5 mg/dL, *p* = 0.004) and A1C levels (nano-curcumin group: 8.18 ± 1.96%; placebo group: 9.22 ± 1.72%, *p* < 0.001) were observed at the end of the study in the nano-curcumin group compared to the placebo group [22].

Adibian et al., performed a randomized, double-blind, placebo-controlled clinical trial to study the effect of curcuminoid administration on 53 patients with T2DM and a previous indication of an intake of oral hypoglycemic agents. Patients were assigned to receive a combination of curcuminoids (1.5 g/day: curcumin, demethoxycurcumin, bis-demethoxy curcumin, and *Curcuma longa* oil) or a placebo for 10 weeks. A significant difference in the change in FPG levels was found in the treatment group compared to the placebo group (*p* = 0.027) [84].

Recently, Shafabakhsh et al., conducted a randomized, double-blind, placebo-controlled clinical trial on 60 T2DM participants to evaluate the effect of nano-curcumin compared to a placebo. Nano-curcumin from Exir Nano Sina Company was used in this clinical trial. Subjects were randomized to receive nano-curcumin (80 mg/day) or a placebo for 12 weeks. A significant reduction in FPG levels (−19.68 mg/dL [95% IC: −33.48, −5.88], *p* < 0.05) was reported between the nano-curcumin and placebo groups. At the end of the intervention period, FPG levels (139.8 ± 40.2 mg/dL vs. 119.1 ± 38.0 mg/dL, *p* < 0.05) also significantly decreased in the nano-curcumin group. Nevertheless, no significant differences were observed in HOMA-IR and the quantitative insulin sensitivity check index with nano-curcumin administration [85].

Hodaei et al. assessed the effect of the administration of curcuminoids on 53 T2DM patients. In this randomized, double-blind, placebo-controlled study, patients received a combination of curcuminoids (1.5 g/day: curcumin, demethoxycurcumin, bis-demethoxy curcumin, and *Curcuma longa* oil) or a placebo for 10 weeks. A significant difference in the mean change in FPG levels (curcuminoids group: −7 ± 2 mg/dL; placebo group: 3 ± 0.2 mg/dL, *p* = 0.02) was observed at the end of the study in the curcuminoid group compared to the placebo group. However, no significant changes were found in insulin levels, A1C levels, homeostasis model of assessment of β cells (HOMA-β), and HOMA-IR [86].

Adab et al. carried out a randomized, double-blind, placebo-controlled clinical trial on 80 hyperlipidemic T2DM patients. Subjects received either rhizome *Curcuma longa* powder (2.1 g/day) or a placebo for 8 weeks. No significant changes were observed in FPG, A1C, HOMA-IR, and insulin levels [87].

Chuengsamarn et al. conducted a randomized, double-blind, placebo-controlled clinical trial on 240 patients with prediabetes. Subjects were randomly allocated to receive a combination of curcuminoids (1500 mg/day: demethoxycurcumin and bisdemethoxycurcumin) or a placebo for 9 months. The study reported significant decreases in FPG levels (curcuminoid group: 86.47 (73–122) mg/dL; placebo group: 108.21 (80–138) mg/dL, *p* < 0.01), PPG levels (curcuminoid group: 123.35 (75–178) mg/dL; placebo group: 155.09 (90–290) mg/dL, *p* < 0.01), A1C levels (curcuminoids group: 5.60 (4.9–6.8)%; placebo group: 6.02 (5.2–7.5)%, *p* < 0.01), and HOMA-IR (curcuminoids group: 3.22 (0.4–11); placebo group: 4.08 (0.3–16.6), *p* < 0.001) at the end of the study in the curcuminoid group compared to the placebo group. In addition, significant results were reported in the development of T2DM as none of the patients in the curcumin group progressed to T2DM compared to the placebo group (19 patients, 16.4%) (*p* < 0.001) [88].

**Table 3:** *Curcuma longa*: clinical evidence in type 2 diabetes mellitus.

Medicinal Plant	Sex/Age (Range or Mean)	T2DM Inclusion Criteria	Blinding/ Randomization	Intervention	Results	Reference
Curcuminoids from *Curcuma longa*	Women and men 18–65 years	FPG ≥ 7.0 mmol/L 2-h PG during OGTT ≥ 11.1 mmol/L	Double-blind/randomized (block randomization with random numbers generated by SPSS)	300 mg/day for 3 months	↓ FPG ↓ A1C ↓ HOMA-IR	[20]
Curcumin from *Curcuma longa*	Women and men >18 years	American Diabetes Association: FPG ≥ 126 mg/dL 2-h PG during OGTT > 200 mg/dL	Double-blind/randomized (random numbers generated by a computer software)	80 mg/day for 3 months	↓ FPG ↓ A1C	[21]
Curcumin from *Curcuma longa*	Women and men 30–60 years	Diagnostic criteria not reported	Double-blind/randomized (block randomization)	80 mg/day for 8 weeks	↓ FPG ↓ A1C	[22]
Curcuminoids from *Curcuma longa*	Women and men 40–70 years	American Diabetes Association	Double-blind/randomized (block randomization)	1.5 g/day for 10 weeks	↓ FPG	[84]
Curcumin from *Curcuma longa*	Women and men 18–80 years	Diagnostic criteria not reported	Double-blind/randomized (computer-generated random numbers)	80 mg/day for 12 weeks	↓ FPG ↔ HOMA-IR ↔ QUICKI	[85]
Curcuminoids from *Curcuma longa*	Women and men 40–70 years	Diagnostic criteria not reported	Double-blind/randomized (block randomization)	1.5 g/day for 10 weeks	↓ FPG ↔ fasting insulin ↔ A1C ↔ HOMA-β ↔ HOMA-IR	[86]
*Curcuma longa* (rhizome powder)	Women and men 30–70 years	FPG < 200 mg/dL A1C > 6.0%	Double-blind/randomized (block randomization)	2.1 g/day for 8 weeks	↔ FPG ↔ A1C ↔ HOMA-IR ↔ fasting insulin	[87]
Curcuminoids from *Curcuma longa*	Women and men ≥35 years	American Diabetes Association: FPG ≥ 100 mg/dL and ≤125 mg/dL A1C ≥ 5.7% and ≤6.4% 2-h PG during OGTT ≥ 140 mg/dL and ≤199 mg/d	Double-blind/randomized (computer-generated random numbers)	1500 mg/day for 9 months (prediabetic patients)	↓ FPG ↓ PPG ↓ A1C ↓ HOMA-IR	[88]

T2DM: type 2 diabetes mellitus; FPG: fasting plasma glucose; A1C: glycated hemoglobin A1c; HOMA-IR: homeostasis model assessment of insulin resistance; QUICKI: quantitative insulin sensitivity check index; HOMA-β: homeostatic model assessment of β cells; PPG: postprandial plasma glucose; PG: plasma glucose; OGTT: oral glucose tolerance test. Parameter changes: ↓: significant decrease; ↑: significant increase; ↔: unchanged.

### 2.4. Berberine

Berberine is a yellow, naturally benzylisoquinoline alkaloid which is the main bioactive compound of *Rhizoma Coptidis*, a popular Chinese herbal medicine [89]. Berberine can be found in roots, rhizomes, stems, and bark of diverse species of the Ranuncolaceae family, including *Berberis vulgaris* (barberry), *Berberis aristata* (tree turmeric), *Copti chinensi* (Chinese goldthread), *Coptis trifolia* (American goldthread), and *Hydrastis canadensis* (goldenseal) [90]. There are different chemical forms of berberine: berberine hydrochloride, berberine sulfate, berberine citrate, or phosphate). Among these, berberine hydrochloride is the most common form [91].

According to data published so far, berberine can possibly modulate glucose metabolism through different mechanisms and signal pathways (Figure 1). In human cultured preadipocytes, berberine enhanced insulin sensitivity by inhibiting fat accumulation and modulating adipokine secretion [92]. In other reports related to insulin sensitivity, it has been demonstrated that in cultured human hepatoma cells, treatment with berberine raised insulin receptor mRNA and protein expression, which entailed an increase in insulin sensitivity. This mechanism was also confirmed in diabetic rats [23]. It has been proposed that AMPK mediates the metabolic activities of berberine. In 3T3-L1 adipocytes and L6 cells, it has been shown that berberine led to an activation of AMPK and the enhancement of glycolysis [93].

Another mechanism of action proposed is related to GLP-1 secretion. A study in normal rats and NCI-H716 cells reported that berberine stimulated GLP-1 secretion and promoted GLP-1 biosynthesis [94]. Berberine also exerts its hypoglycemic action by inhibiting intestinal disaccharidases. In diabetic rats, berberine inhibited disaccharidase activities and sucrase–isomaltase complex mRNA expression. Caco-2 cells have been used as an in vitro model to study α-glucosidase inhibitors. In these cells, berberine also suppressed disaccharidase activity [95].

Evidence suggests that gut microbiota composition is linked to T2DM [96]. It has been reported that berberine exerts antidiabetic effects through modulating gut microbiota composition. In C57BL/6 mice, berberine reversed the suppressed level of short-chain fatty acids and increased the ratio of Firmicutes and Bacteroidetes [97].

Animal studies have been conducted to demonstrate the hypoglycemic effect of berberine. In a study with STZ-induced type 2 diabetic rats, berberine treatment (75 or 150 mg/kg) for 15 days significantly decreased fasting blood glucose and insulin resistance compared to the untreated rats [23]. STZ- induced diabetic rats treated with berberine (380 mg/kg) for 4 weeks showed a significant reduction in FPG levels and HOMA-IR compared to the control group [24]. In another study with STZ- induced diabetic rats, the intervention with berberine (380 mg/kg) for 5 weeks significantly decreased fasting blood glucose levels compared to the untreated control group [98].

In a study realized in type 2 diabetic Goto-Kakizaki rats, berberine chloride hydrate administration (200 mg/kg) for 8 weeks led to a marked and significant reduction of fasting blood glucose levels and HOMA-IR in comparison with the control group [99]. Recently, a significant decrease in fasting blood glucose levels and HOMA-IR has been reported in STZ-induced diabetic rats compared to the control group after treatment with berberine (187.5 mg/kg) for 6 weeks [100].

After 7 weeks of berberine supplementation (100 mg/kg), a significant reduction in fasting blood glucose levels and an improvement in oral glucose tolerance were observed in STZ-induced diabetic rats compared to the control group [101]. The effect of berberine has also been evaluated in the prevention of T2DM. In Zucker diabetic fatty rats, berberine hydrochloride administration (100 mg/kg) for 3 weeks resulted in a significant decrease in fasting blood glucose levels, oral glucose tolerance, and HOMA-IR in comparison with the IGT group [102].

Regarding clinical trials, several studies have been carried out to examine the antidiabetic effect of berberine on patients with T2DM (Table 4). Yin et al. carried out a pilot randomized study to determine the efficacy of berberine hydrochloride in the treatment of T2DM patients. The study involved two sub-studies. In study A, 36 patients with newly diagnosed T2DM were assigned to receive berberine or metformin (1.5 g/day) for 3 months. After berberine administration, significant decreases in FPG levels (10.6 ± 0.9 mmol/L vs. 6.9 ± 0.5 mmol/L, *p* < 0.01), PPG levels (19.8 ± 1.7 vs. 11.1 ± 0.9 mmol/L, *p* < 0.01), and A1C levels (9.5 ± 0.5% vs. 7.5 ± 0.4%, *p* < 0.01) were reported. Fasting insulin and postprandial insulin levels were not significantly modified. In study B, 48 adults with poorly controlled T2DM treated with sulfonylureas, metformin, acarbose, or insulin therapy alone or with a combination were allocated to receive berberine (1.5 g/d) for 3 months. Berberine significantly decreased FPG levels (9.6 ± 0.4 mmol/L vs. 7.6 ± 0.3 mmol/L, *p* < 0.001), PPG levels (14.8 ± 0.7 mmol/L vs. 9.7 ± 0.9 mmol/L, *p* < 0.001), A1C levels (8.1 ± 0.2% vs. 7.3 ± 0.3%, *p* < 0.001), and HOMA-IR (15.2 ± 1.6 mmol/L vs. 8.4 ± 1.8 mmol/L, *p* < 0.001) [25].

Zhang et al. conducted a randomized clinical trial with 97 T2DM patients. Patients were assigned to receive berberine hydrochloride (1 g/d), metformin (1.5 g/d), or rosiglitazone (4 mg/d). After 2 months of intervention, berberine significantly lowered FPG levels (10.4 ± 0.4 mmol/L vs. 7.7 ± 0.3 mmol/L, *p* < 0.001) and A1C levels (8.3 ± 0.3% vs. 6.8 ± 0.2%, *p* < 0.001). The decrease found in FPG levels and A1C levels in the berberine group was similar to those of metformin and rosiglitazone [26].

The effect of berberine has also been compared to placebo by Rashidi et at. in a randomized, double-blind, placebo-controlled clinical trial of 84 patients with T2DM. In addition to their previous medications, the intervention consisted of the administration of berberine (1 g/day) or placebo for 4 weeks. After berberine administration, significant decreases were observed in FPG levels (192 ± 59.6 mg/dL vs. 167.7 ± 51.8 mg/dL, *p* < 0.001) and PPG levels (266.1 ± 93.7 mg/dL vs. 222.5 ± 76 mg/dL, *p* < 0.001). In addition, HOMA-β function significantly increased (31.2 ± 16.9 vs. 45.7 ± 30.1, *p* < 0.001). Fasting insulin levels and HOMA-IR were not significantly modified after berberine administration [27].

Zhang et al. evaluated the efficacy of berberine on 116 newly diagnosed T2DM patients with dyslipidemia without previous treatment in a randomized, double-blind, placebo-controlled trial. Patients received berberine (1 g/day) or a placebo for 3 months. After berberine administration, significant changes were found in FPG levels (7.0 ± 0.8 mmol/L vs. 5.6 ± 0.9 mmol/L, *p* = 0.000), PPG levels (12.0 ± 2.7 mmol/L vs. 8.9 ± 2.8 mmol/L, *p* = 0.000), A1C levels (7.5 ± 1.0% vs. 6.6 ± 0.7%, *p* = 0.000), and HOMA-IR (3.90 ± 3.20 vs. 2.44 ± 1.67, *p* = 0.010). Fasting serum insulin levels and post-load serum insulin levels were not modified [103].

A randomized, double-blind, placebo-controlled trial was carried out by the same research group on 409 newly diagnosed T2D patients. Participants were randomly assigned to receive berberine alone (7.2 g/day), probiotics (4 g/day) plus berberine, probiotics alone, or a placebo for 12 weeks. The results showed that changes in A1C levels in the probiotics plus berberine (least-squares mean (95% CI), −1.04(−1.19, −0.89)%) and berberine alone groups (−0.99(−1.16, −0.83)%) were significantly greater than in the placebo and probiotics-alone groups (−0.59(−0.75, −0.44)%, −0.53(−0.68, −0.37)%, *p* < 0.001). HOMA-IR significantly decreased (*p* not reported) by probiotics plus berberine [104].

Berberine has also been studied in combination with other substances. Harrison et al. evaluated in a randomized, double-blind, placebo-controlled trial the effect of berberine ursodeoxycholate, an ionic salt of berberine and ursodeoxycholic acid (2 g/day and 1 g/day), in comparison with placebo on 100 patients with presumed non-alcoholic steatohepatitis and T2MD drug naïve patients for a period of 18 weeks. After the intervention with berberine ursodeoxycholate, A1C levels significantly decreased (*p* not reported) by 0.6% (2 g/day) and 0.3% (1 g/day). No significant changes were reported in FPG levels, insulin levels, and HOMA-IR [105].

Di Pierro et al. assessed the combination of berberine and silymarin in addition to an oral hypoglycemic regimen in a pilot study of 26 T2DM patients. All patients received Berberol^®^ (588 mg of *Berberine aristata* and 105 mg of *Silybum marianum*) for 90 days. Significant decreases were observed in A1C levels (8.0 ± 0.82% vs. 7.15 ± 1.09%, *p* = 0.003), and HOMA-IR (6.9 ± 3.6 vs. 5.1 ± 3.6, *p* = 0.04). There was not a significant change in FPG levels [106].

**Table 4:** Berberine: clinical evidence in type 2 diabetes mellitus.

Medicinal Plant	Sex/Age (Range or Mean)	T2DM Inclusion Criteria	Blinding/ Randomization	Intervention	Results	Reference
Berberine (hydrochloride)	Women and men 25–75 years	FPG > 7.0 mmol/L A1C > 7.0%	Blinding not reported)/ randomized (method not reported)	Study A 1.5 g/day for 3 months Study B 1.5 g/day for 3 months	Study A ↓ FPG ↓ PPG ↓ A1C ↔ fasting insulin ↔ postprandial insulin Study B ↓ FPG ↓ PPG ↓ A1C ↓ HOMA-IR	[25]
Berberine (hydrochloride)	Women and men 57 ± 8 years	FPG ≥ 7.0 mmol/L 2-h PG during OGTT ≥ 11.1 mmol/L	Blinding not reported)/ randomized (method not reported)	1 g/day for 2 months	↓ FBG ↓ A1C	[26]
Berberine	Women and men 30–65 years old	American Diabetes Association: FPG ≥ 126 mg/dL and ≤200 mg/dL A1C ≥ 7% and ≤8.5%	Double-blind/ randomized (block randomization)	1 g/day for 4 weeks	↓ FPB ↓ PPG ↑ HOMA-β ↔ fasting insulin ↔ HOMA-IR	[27]
Berberine	Women and men 25–70 years	World Health Organization: FPG ≥ 7 mmol/L and <8 mmol/L 2-h PG during OGTT ≥ 11.1 mmol/L	Double-blind/ randomization (block randomization)	1 g/day for 3 months	↓ FPG ↓ PPG ↓ A1C ↓ HOMA-IR ↔ fasting insulin ↔ postprandial insulin	[103]
Berberine alone Berberine plus probiotics	Women and men 20–70 years	World Health Organization: FPG ≥ 7.0 mmol/L and ≤13.3 mmol/L A1C ≥ 6.5% and ≤10.0%	Double-blind/ randomization (block randomization)	7.2 g/day for 12 weeks	↓ A1C ↓ HOMA-IR	[104]
Berberine plus ursodeoxycholic acid	Women and men 26–75 years	A1C ≥ 5.1% and ≤9.4%	Double blind/ randomization (block randomization)	1 g/day or 2 g/day for 18 weeks	↓ A1C ↔ FPG ↔ fasting insulin ↔ HOMA-IR	[105]
Berberine (*Berberis aristata*) plus *Silybum marianum*	Women and men 25–75 years	A1C ≥ 7.5% and ≤9.5%	Blinding not reported/ randomization not reported	588 mg for 90 days	↓ A1C ↓ HOMA-IR ↔ FPG	[106]

T2DM: type 2 diabetes mellitus; FPG: fasting plasma glucose; PPG: postprandial plasma glucose; A1C: glycated hemoglobin A1c; HOMA-IR: homeostasis model assessment of insulin resistance; HOMA-β: homeostasis model of assessment of β cells; PG: plasma glucose; OGTT: oral glucose tolerance test. Parameter changes: ↓: significant decrease; ↑: significant increase; ↔: unchanged.

### 2.5. Momordica charantia

*Momordica charantia,* commonly known as bitter melon, is a medicinal plant that belongs to the Cucurbitaceae family. *Momordica charantia* grows in tropical and subtropical regions and is widely used in traditional medicine in Asia, India, South America, East Africa, and the Caribbean [107]. Different bioactive compounds such as charantin, polypeptide-p, cucurbitane glycosides, and phenolic compounds have been described in *Momordica charantia.* The fruit and seeds of *Momordica charantia* have been studied for their hypoglycemic properties in animal models and clinical studies [108,109].

There are multiple mechanisms responsible for the antidiabetic activity of *Momordica charantia* (Figure 1). A significant decrease in maltase and lactase activities was observed in STZ-induced diabetic rats treated with *Momordica charantia* powder fruit [110]. An inhibitory effect on α-glucosidase by cucurbitane-type triterpene glycosides isolated from *Momordica charantia* fruit has also been reported [111].

In STZ-induced diabetic rats, *Momordica charantia* juice administration significantly reduced Na + -dependent glucose uptake by the intestinal mucosa [112].

It has also been proposed that *Momordica charantia* can improve glucose metabolism by regulating key enzymes. Glucokinase, hexokinase, and phosphofructokinase activities increased in STZ-induced diabetic mice treated with *Momordica charantia*, thus facilitating glucose oxidation. Stimulation of glycogenesis was also reported in this study evidenced by an increment in hepatic and muscle glycogen content [113].

Administration of *Momordica charantia* fruit extract to alloxan-induced diabetic rats resulted in a significant inhibitory effect on glycogenolysis [114].

Suppression of hepatic gluconeogenic enzymes, glucose-6-phosphatase and fructose-1,6-bisphosphatase, has also been demonstrated in STZ-induced diabetic rats after treatment with an ethanolic extract of *Momordica charantia* [115].

There is also evidence that *Momordica charantia* can improve insulin sensitivity. Triterpenoids isolated from *Momordica charantia* stimulated GLUT-4 translocation to the cell membrane in L6 myotubes and 3T3-L1 adipocytes; activation of the AMPK signaling pathway was associated with this effect [116]. In the skeletal muscle of rats fed a high-fructose diet, *Momordica charantia* fruit extract administration improved protein and mRNA expression of GLUT-4. The same study reported that *Momordica charantia* increased the expression of PPARγ in white adipose tissue [117]. A similar result was observed in C57BL/6J mice fed with an HFD; *Momordica charantia* fruit extract increased mRNA expression of PPARγ in adipose tissue [118].

Furthermore, in high-fat diet-fed Wistar rats, *Momordica charantia* fruit extract supplementation increased skeletal muscle insulin-stimulated IRS-1 tyrosine phosphorylation [119]. Protein tyrosine phosphatase 1B (PTP1B) is the physiological antagonist of the insulin signaling pathway. The saponin and lipid fractions of *Momordica charantia* fruit reduced PTP1B activity in skeletal muscle in db/db mice [120].

Pertaining to insulin secretion, the aqueous extract of *Momordica charantia* fruit significantly increased GLP-1 levels in diabetic rats. GLP-1 is an incretin hormone that enhances insulin secretion through different mechanisms [121]. Moreover, treatment of alloxan-induced and STZ-induced diabetic rats with *Momordica charantia* fruit extract increased the number of β-cells, islet size, and total β-cell area and induced the regeneration of β-cells in pancreatic islets [122,123,124].

The antidiabetic activity of *Momordica charantia* has also been reported in animal models. In STZ-induced diabetic rats, administration of three different doses of *Momordica charantia* (2, 5, and 10% of the standard diet) for 12 weeks significantly reduced fasting blood glucose levels compared to untreated diabetic rats [28]. A significant reduction in blood glucose levels was also observed in STZ-induced diabetic rats after treatment with *Momordica charantia* nanoparticles (50 mg/kg) and *Momordica charantia* aqueous extract (100 mg/kg) for 11 days [29].

Treatment with different doses (150 mg/kg, 300 mg/kg, and 600 mg/kg) of *Momordica charantia* fruit extract to alloxan-induced diabetic rats for 30 days reduced blood glucose levels and glycosylated hemoglobin levels. The increase in blood sugar levels in glucose-loaded rats was also significantly inhibited [114].

Similar results were observed in another study in alloxan-induced diabetic rats. Administration of methanolic extract of *Momordica charantia* (80 mg/kg) for 30 days significantly improved the levels of fasting blood glucose, glycated hemoglobin, and insulin [125].

A significant increase in serum insulin levels was also reported in STZ-induced diabetic rats that received *Momordica charantia* (250 mg/kg and 500 mg/kg) for four weeks compared to the diabetic control group. Significant reductions in blood glucose levels after an OGTT and HOMA-IR were also observed when compared to diabetic control rats [126].

Regarding clinical trials, several studies in T2DM patients have shown a positive effect of *Momordica charantia* administration on glycemic control parameters (Table 5). Lim et al. carried out a randomized, double-blind clinical trial of 40 T2DM patients. Patients were assigned to receive one of three single doses of *Momordica charantia* from dried leaves (60, 80, or 100 mg/kg/day) or a placebo. Insulin concentrations were determined at 0, 15, 30 min, 1, 2, and 4 h after the given dose. An increase in plasma insulin levels was observed in the three *Momordica charantia* groups compared to the placebo group. A significant increment in insulin secretion was observed with the dose of 100 mg/kg/day during the initial 15-min post-meal interval compared to the other *Momordica charantia* doses and the placebo group [30].

Cortez-Navarrete et al. also assessed the effect of *Momordica charantia* administration on insulin secretion and insulin sensitivity. A randomized, double-blind, placebo-controlled clinical study was conducted on 24 patients with newly diagnosed T2DM without pharmacological therapy. Patients were randomized to receive *Momordica charantia* fruit powder (2 g/day) or a placebo for 12 weeks.

Significant increases in total insulin secretion (0.29 ± 0.18 vs. 0.41 ± 0.29, *p* = 0.028) and in the first phase of insulin secretion (557.8 ± 645.6 vs. 1135.7 ± 725.0, *p* = 0.043) were reported in the *Momordica charantia* group. A1C levels (7.8 ± 0.8% vs. 7.1 ± 1.3%, *p* < 0.05) and PPG levels (17.1 ± 3.7 mmol/L vs. 13.2 ± 4.3 mmol/L, *p* < 0.01) also significantly decreased after MC administration. No significant changes were reported in FPG levels and insulin sensitivity after the intervention with *Momordica charantia* [31].

The effect of *Momordica charantia* administration on HOMA-IR, HOMA-β, and glycemic control parameters was evaluated by Kim et al. in a randomized, double-blind, placebo-controlled study. Ninety T2DM patients were randomly assigned to receive *Momordica charantia* fruit extract (2.38 g/day) or a placebo for 12 weeks. Subjects could be under treatment with antidiabetic drugs, excluding alpha-glucosidase inhibitors. HOMA-IR (2.4 (1.3–3.5) vs. 1.8 (1.3–2.8), *p* = 0.017) and FPG levels (145.9 ± 34.5 mg/dL vs. 140.5 ± 31.9 mg/dL, *p* = 0.014) significantly decreased from baseline to endpoint in the *Momordica charantia* group. However, no significant changes in A1C and HOMA-β were reported [32].

The hypoglycemic effect of *Momordica charantia* has been compared with antidiabetic drugs. Rahman et al. performed a randomized, double-blind, parallel-group trial on 95 T2DM patients. Subjects were randomly allocated to two different doses of *Momordica charantia* fruit powder (group I: 2 g/day and group II: 4 g/day) or glibenclamide (group III: 5 mg/day) for 10 weeks. Significant decreases in the mean levels of A1C (group I: 8.25 ± 0.70% vs. 7.40 ± 0.50%, *p* ≤ 0.05; group II: 8.30 ± 0.55% vs. 7.15 ± 0.60%, *p* ≤ 0.02; group III: 8.45 ± 0.60% vs. 6.90 ± 0.75%, *p* < 0.005) and FPG levels (group I: 146 ± 13.40 mg/dL vs. 133.70 ± 11.50 mg/dL, *p* ≤ 0.05; group II: 141.60 ± 15.20 mg/dL vs. 126.40 ± 11.90 mg/dL, *p* < 0.04; group III: 143.50 ± 18.40 mg/dL vs. 117 ± 10.30 mg/dL, *p* < 0.003) were observed in the three intervention groups. There were no significant changes in PPG after the *Momordica charantia* administration [127].

Suthar et al. conducted an open-label, randomized, active-controlled trial of 85 T2DM patients who were on a stable regimen with antidiabetic medication. Patients were randomly assigned to dry *Momordica charantia* fruit juice powder (1.2 g/day) or a placebo for 90 days. FPG and PPG levels (*p* = 0.013 and *p* = 0.002, respectively) significantly decreased in the *Momordica charantia* group compared to the placebo group. The reduction in A1C did not reach statistical significance [128].

The antidiabetic effect of *Momordica charantia* has also been evaluated in a prediabetes population. Krawinkel et al. carried out a randomized, placebo-controlled, single-blind, crossover clinical trial of 52 prediabetic patients. Subjects were randomly allocated to receive *Momordica charantia* fruit powder (2.5 g/day) for 8 weeks, followed by a 4-week washout period. A significant difference in the change of FPG levels was observed in the *Momordica charantia* group (−0.31 mmol/L, *p* ≤ 0.01) compared to the placebo group. No significant changes in FPG, A1C, and insulin levels were reported after *Momordica charantia* administration [129].

**Table 5:** *Momordica charantia*: clinical evidence in type 2 diabetes mellitus.

Medicinal Plant	Sex/Age (Range or Mean)	T2DM Inclusion Criteria	Blinding/ Randomization	Intervention	Results	Reference
*Momordica charantia* (dried leaves)	Women and men 21–65 years	American Diabetes Association: FPG ≥ 126 mg/dL and ≤205 mg/dL A1C ≥ 6.5% and ≤9.0%	Double-blind/ randomized (method not reported)	100 mg/kg/day (single dose)	↑ insulin secretion	[30]
*Momordica charantia* (fruit powder)	Women and men 35–60 years	American Diabetes Association: FPG < 11.6 mmol/L A1C ≥ 7% and ≤9%	Double-blind/ randomized (random number list)	2 g/day for 12 weeks	↑ insulin secretion ↓ PPG ↓ A1C ↔ FPG ↔ insulin sensitivity	[31]
*Momordica charantia* (fruit extract)	Women and men 20–70 years	A1C ≤ 7.5%	Double-blind/ randomized (block randomization)	2.38 g/day for 12 weeks	↓ HOMA-IR ↓ FPG ↔ A1C ↔ HOMA-β	[32]
*Momordica charantia* (fruit powder)	Women and men 30–70 years	World health Organization: FPG ≥ 126 mg/dL and ≤240 mg/dL	Double-blind/ randomized (method not reported)	2 g/day and 4 g/day for 10 weeks	↓ A1C ↓ FPG ↔ PPG	[127]
*Momordica charantia* (fruit juice powder)	Women and men 30–70 years	FPG ≥ 110 mg/dL and <250 mg/dL A1C > 7% and <10%	Open-label/ randomized (block randomization)	1.2 g/day for 90 days	↓ FPG ↓ PPG ↔ A1C	[128]
*Momordica charantia* (fruit powder)	Women and men 30–65 years	FPG ≥ 5.6 mmol/L and ≤6.9 mmol/L A1C ≥ 5.7% and ≤7.5%	Single-blind/ randomized (Mersenne Twister random number generator)	2.5 g/day for 8 weeks (prediabetic patients)	↓ FPG ↔ A1C ↔ fasting insulin	[129]

T2DM: type 2 diabetes mellitus; PPG: postprandial plasma glucose; A1C: glycated hemoglobin A1c; FPG: fasting plasma glucose; HOMA-IR: homeostasis model assessment of insulin resistance; HOMA-β: homeostasis model of assessment of β cells. Parameter changes: ↓: significant decrease; ↑: significant increase; ↔: unchanged.

## 3. Conclusions

The current treatment for T2DM includes a combination of lifestyle modifications and antidiabetic drugs. The long-term administration of antidiabetic drugs is associated with unwanted side effects, toxicity, and high costs. For this reason, it is important to search for safe and effective therapeutic alternatives to synthetic drugs. Medicinal plants have been used for centuries in traditional medicine and represent an alternative or complementary option in the management of T2DM. Fenugreek, cinnamon, *Curcuma longa*, berberine, and *Momordica charantia* have demonstrated antidiabetic effects through different mechanisms of action reported in the literature. Furthermore, evidence from preclinical studies and clinical trials have shown their potential for the treatment of T2DM. However, there are limitations regarding their use in clinical practice. There is a limited number of studies that evaluate the possible interactions with antidiabetic medication; therefore, research should focus on evaluating the frequency and nature of these interactions to determine their potential health benefits and risks. At the same time, the characterization and standardization of bioactive compounds should be a focal point of research in order to assess their bioavailability. In addition, long-term side effects and toxicity should be monitored.

We consider that the medicinal plants presented in this review have potential to be used as an alternative or complementary treatment for T2DM. However, further clinical trials with adequate sample sizes, randomized, placebo controlled, and with longer intervention periods are needed to confirm their safety and efficacy.

## Data Availability

Not applicable.
